# Supplementary material for: Quantifying Cross-Attention Interaction in Transformers for Interpreting TCR-pMHC Binding
Source: ArXiv. 2026 Mar 5:arXiv:2507.03197v3. Preprint. [Version 3] (PMC12976929)
Supplement: Supplement 1 [file NIHPP2507.03197v3-supplement-1.pdf]

A Supplementary Material

A.1 Post-hoc Explanation Methods

A variety of explainable AI (XAI) methods have been developed to interpret deep learning models (Saranya & Subhashini, 2023). These methods fall into two broad categories: explain-by-design, which integrates interpretability into the model architecture (Dwivedi et al., 2023), and post-hoc, which analyzes model behavior after training (Kenny et al., 2021). Post-hoc approaches offer a promising avenue for interpreting TCR-pMHC models and uncovering the underlying factors driving binding predictions. Several families of post-hoc methods have been proposed, including:

- The Class Activation Map (CAM) (e.g., CAM (Zhou et al., 2016), GradCAM (Selvaraju et al., 2017), GradCAM++ (Chattopadhay et al., 2018))
- Layer-wise Relevance Propagation (LRP) (e.g., LRP (Binder et al., 2016), Partial LRP (Voita et al., 2019), Conservative LRP (Ali et al., 2022), AttnLRP (Achtibat et al., 2024))
- Attention-based methods (e.g., Raw Attention (Wiegreffe & Pinter, 2019), Attention Rollout (Abnar & Zuidema, 2020), AttCAT (Qiang et al., 2022))
- Model-specific hybrid methods (e.g., TokenTM (Wu et al., 2024a), GAE (Chefer et al., 2021))

These techniques have been successfully applied to TCR-pMHC models. For example, TEPCAM interpret a attention-CNN with attention map (Chen et al., 2024), while TCR-BERT relies on attention weight analysis for interpretability (Wu et al., 2024b). These efforts have revealed structural determinants of TCR-pMHC binding. However, existing post-hoc methods primarily support encoder-only or co-attention mechanisms (Chefer et al., 2021), limiting their applicability to modern encoder-decoder models, which consists of cross-attention. This poses a major barrier to understanding how such models capture TCR-pMHC interactions.

A.2 Class Activation Maps

Class Activation Map (CAM)-based methods have achieved significant success in explaining Convolutional Neural Networks (CNNs) by generating class-discriminative localization maps. Grad-CAM (Selvaraju et al., 2017), one of the most effective CAM methods, leverages the gradient of the class score L^c with respect to the feature maps F_d from the last convolutional layer. These gradients are used to compute importance weights for each feature map channel, enabling spatial localization of the regions most relevant for class c. The importance weight w_d^c for feature map F_d is computed as:

$$w_d^c = \mathbb{E}\left(\frac{\partial L^c}{\partial F_d}\right),$$

where $\mathbb{E}$ denotes global average and w_d^c represents the global average pooled gradient for feature map F_d. The final CAM is then computed as a weighted sum over channels, followed by a ReLU activation:

$$\text{GradCAM}^c = \text{ReLU}\left(\sum_d w_d^c F_d\right).$$

The resulting heatmap is upsampled to the input resolution to highlight input regions most relevant to the prediction for class c.

A.2.1 Attention Rollout

CAM-based approaches are primarily designed for CNNs. To interpret transformer-based models, Attention Rollout was proposed by Abnar & Zuidema (2020), which estimates the flow of attention across layers. This method computes how information propagates through the self-attention mechanism across layers. Given the raw attention weights W_l^A for layer l, the augmented attention matrix is defined as

$$A_l = \frac{1}{2}(W_l^A + I),$$

where I is the identity matrix, modeling the residual connection. The cumulative attention, or rollout, is then computed recursively:

$$R_l = \begin{cases} A_l R_{l-1}, & \text{if } l > 0 \\ A_l, & \text{if } l = 0 \end{cases},$$

capturing the total attention contribution from input tokens through layer l.

A.3 TCR-pMHC Binding Prediction

T cells are important component of our immune system, which can be mainly catogorized in two CD8+ and CD4+ T cells. CD8+ T cells are initiated through the Major Histocompatibility Complex I (MHCI) pathway, while CD4+ T cells are initiated through the MHCII pathway. Epitope prediction for CD8+ T cells has had remarkable success, while the mechanisms of CD4+ T cell response are less understood. T cell immune response can be viewed as consisting of two stages of recognition. In the first stage, a antigen is taken up by antigen-presenting cells (APCs), where it undergoes joint processing (i.e., cleavage) and binding to Major Histocompatibility Complex II (MHCII) molecules. Peptide-MHC complexes are then presented on the APC cell surface (Davis & Bjorkman, 1988; Neefjes et al., 2011). In the second stage, T cell receptors (TCRs) on T cells "recognize" pMHC complexes and a T cell response is initiated. TCR recognition is mediated by its α and β domains, which consist of variable (V), joining (J), constant (C), and, in the β chain, diversity (D) regions (Bosselut, 2019).

Accurate prediction of T cell responses requires a comprehensive understanding of both of these stages (Peters et al., 2020; Nielsen et al., 2020). Early efforts in the area of computational epitope prediction focused on characterizing peptide-MHCII binding using allele-specific machine learning models (Nielsen et al., 2020) with tools such as SMM (Peters & Sette, 2005; Kim et al., 2009), NetMHC (Lundegaard et al., 2008; Nielsen et al., 2003), NetMHCpan (Hoof et al., 2009; Nielsen et al., 2007), and NetMHCcons (Karosiene et al., 2012). More recent work has focused on modeling antigen processing computationally with the Antigen Processing Likelihood (APL) algorithm (Mettu et al., 2016; Bhattacharya et al., 2023; Li et al., 2024a;b; Charles et al., 2022), which seeks to model the contributions of antigen structure on which peptides are made available for MHCII binding.

Accurately predicting TCR-pMHC binding remains critical for advancing quantitative immunology and adaptive immunity research (Hudson et al., 2023). For this stage of prediction, both unsupervised and supervised methods have been developed (Hudson et al., 2023; 2024). Unsupervised methods process cluster TCR sequencing datasets through dimensionality reduction and clustering (Dash et al., 2017; Glanville et al., 2017) through a carefully chosen similarity metric (e.g., TCRdist3 (Mayer-Blackwell et al., 2021)). These methods cluster TCRs by analyzing their complementarity-determining regions (CDRs) using only TCR sequence data, without requiring binding labels or epitope information (e.g., GIANA (Zhang et al., 2021), ClusTCR (Valkiers et al., 2021), GLIPH2 (Huang et al., 2020) iSMART (Zhang et al., 2020)). The resulting cluster labels serve as the output for each input TCR sequence (Hudson et al., 2024) and are typically analyzed by practitioners to guide and supplement experimental methods. In contrast, supervised machine learning techniques make use of large amounts of TCR-pMHC data for training (Hudson et al., 2023) from databases such as VDJdb (Bagaev et al., 2020), McPAS-TCR (Tickotsky et al., 2017) and the IEDB (Vita et al., 2019). Supervised approaches (e.g. TITAN (Weber et al., 2021), STAPLER (Kwee et al., 2023), ERGO2 (Springer et al., 2021), MixTCRpred (Croce et al., 2024), NetTCR2.2 (Jensen & Nielsen, 2024), TULIP (Meynard-Piganeau et al., 2024)) use a variety of deep learning models providing reasonable performance and generalization capability.

A.3.1 TCR-pMHC Binding Problem Formation

The TCR-pMHC binding prediction problem can be formulated as a classification task: given the TCR alpha (α) and beta (β) chains, an epitope e, and an MHC molecule m, the model predicts whether the pair binds (binder) or does not bind (non-binder). The TCR chains and the epitope are proteins or peptides, typically represented as amino acid sequences. Formally, we define amino acid units as $a \in \mathbb{A}$, where $\mathbb{A}$ is the set of amino acid characters. For a single TCR-pMHC binding case, $\alpha = [a_i^{\alpha}]_{i=1}^{N_\alpha}$, $\beta = [a_i^{\beta}]_{i=1}^{N_\beta}$, and $e = [a_i^{e}]_{i=1}^{N_e}$, with $N_\alpha, N_\beta, N_e \in \mathbb{Z}^+$ representing the sequence lengths. The MHC allele type is denoted by $m \in M$, where M is the set of all MHC alleles. The

pMHC-TCR binding classification is formulated as a conditional probability: $p_{\text{bind}} = P(e|\alpha, \beta, m)$. If $p_{\text{bind}} > t$, where $t \in [0, 1]$, the case is classified as positive, otherwise negative.

A.4 TCR-pMHC Prediction Transformer Models

Transformers (Vaswani et al., 2017), as a successful deep learning models in different areas, have a series of variants such as Bidirectional Encoder Representations from Transformers (BERT) (Devlin et al., 2019) and Generative Pre-training Transformers (GPT) (Radford et al., 2018). These models support multi-sequence inputs and excel in modeling interactions, are well-suited for this task. Because TCR-pMHC interactions are determined by interactions among the TCR α and β chains, epitope, and MHC, several state-of-the-art models, such as TULIP (Meynard-Piganeau et al., 2024) and cross-TCR-interpreter (Koyama et al., 2023), adopt encoder-decoder transformer architectures to learn these complex relationships.

TULIP: TULIP is a transformer-based model with an encoder-decoder architecture designed for TCR-pMHC binding prediction. It operates through three parallel modality processing pipelines, processing CDR3a, CDR3b, and epitope sequences separately (Meynard-Piganeau et al., 2024). The encoders transform the input sequences into feature representations, while the decoders model interactions across different sequences (Devlin et al., 2019; Vaswani et al., 2017). As an auto-regressive generative model, TULIP computes the conditional probability distribution of sequences (e.g., epitope) given others (e.g., CDR3a, CDR3b, and MHC) during training (Meynard-Piganeau et al., 2024). For evaluation, TULIP retains only the epitope stream to produce the binding score. In this setting, the peptide features serve as the query, while the CDR3a and CDR3b features are used as the keys and values in the cross-attention module. To compute gradients for TULIP, we design an amino-acid-wise loss function. The ground truth is derived from the TCR alpha, TCR beta, and epitope sequences. These sequences are first one-hot encoded, and the model's predicted probabilities are compared against them using a negative log-likelihood (NLL) loss. This formulation allows us to attribute importance scores at the amino acid level based on how well the model reconstructs each residue.

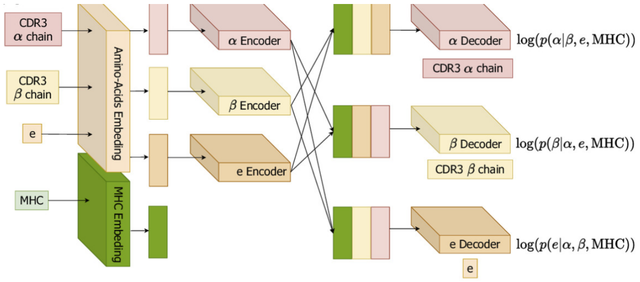

Figure 6: The architecture figure of TULIP model (Meynard-Piganeau et al., 2024).

CrossTCRInterpreter: CrossTCRInterpreter is an encoder-decoder transformer for TCR-pMHC binding prediction (Koyama et al., 2023). It takes the CDR regions of the alpha and beta chains, along with the peptide sequence, as inputs. The CDR alpha and beta chains are concatenated using a colon (:) to form the TCR input. The TCR and peptide sequences are then independently encoded by an encoder module. Subsequently, cross-attention is employed to model the interaction between the two inputs and predict whether the pair represents a binder or a non-binder. We apply a binary classification loss to extract the model gradients.

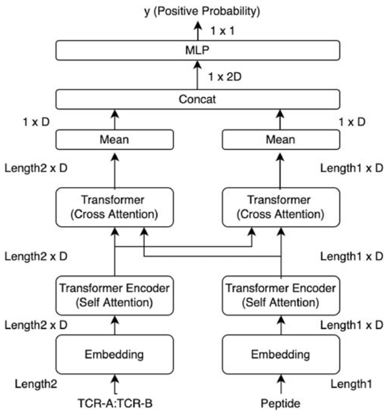

Figure 7: The architecture of CrossTCRInterpreter model (Koyama et al., 2023).

BERTrand: BERTrand is an encoder-only transformer model (Myronov et al., 2023). It takes the TCR beta chain and peptide sequence as inputs. These two sequences are concatenated using a <SEP> token and are processed jointly by a transformer encoder as an integrated input. Similar to CrossTCRInterpreter, BERTrand is a classification model designed to predict whether the TCR-pMHC pair is a binder or a non-binder. We apply a binary classification loss to obtain the model gradients.

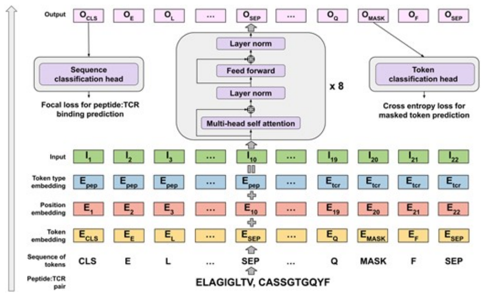

Figure 8: The architecture figure of BERTrand model (Myronov et al., 2023).

A.5 PERTURBATION EXPERIMENTS

We evaluated the robustness of interpretability methods using perturbation-based metrics across varying values of k. Figure 9 presents the comparison results for both AOPC and LOdds across all chains.

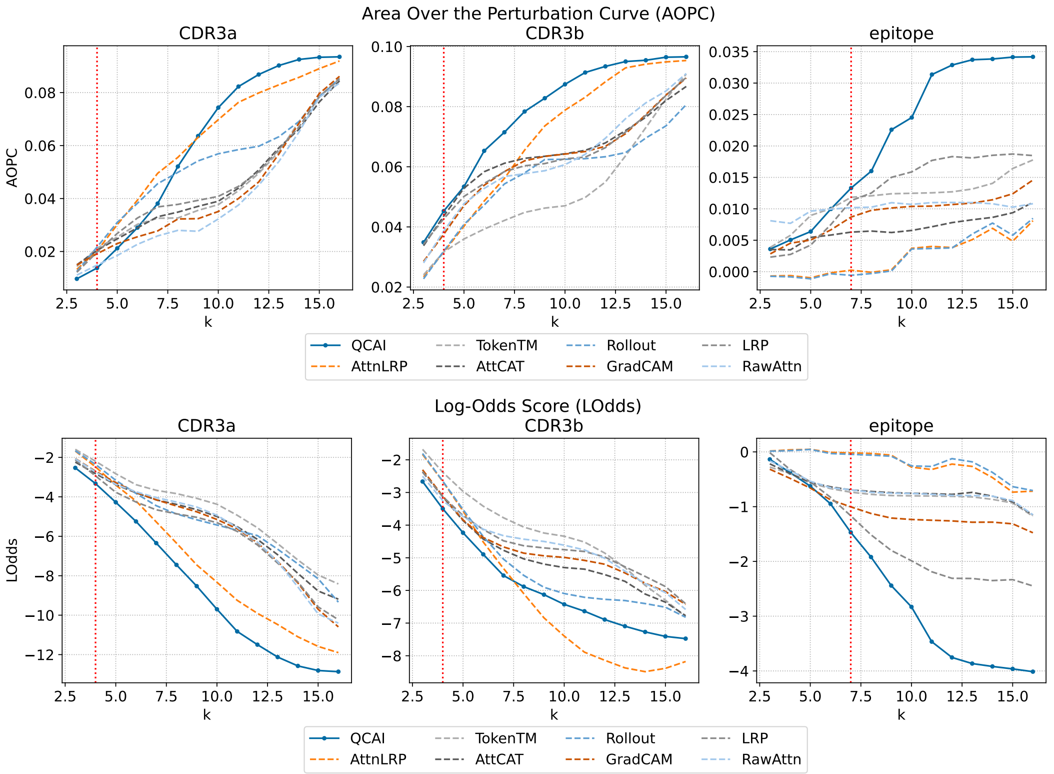

Figure 9: Comparison of Area Over the Perturbation Curve (AOPC) and Comparison of Log-Odds Score (LOdds) across different values of k for all chains.

We also conducted with an integrated dataset that includes data from VDJdb, IEDB and McPAS-TCR. For both AOPCs and LOdds, the thresholds for peptide, CDR3a, and CDR3b are 7, 4, and 4 respectively.

		AOPCs		LOdds	
Chain	Method	TCR-XAI	Integrated	TCR-XAI	Integrated
peptide	QCAI	0.014	**0.036**	-1.62	**-0.84**
peptide	TokenTM	0.013	0.026	-0.77	0.09
peptide	AttnLRP	0.012	0.026	-0.42	-0.50
CDR3a	QCAI	0.014	0.020	-3.50	**-2.63**
CDR3a	TokenTM	0.021	0.020	-2.43	-2.35
CDR3a	AttnLRP	0.020	0.020	-2.72	-2.44
CDR3b	QCAI	0.048	**0.027**	-3.61	**-3.08**
CDR3b	TokenTM	0.033	0.025	-2.53	-2.78
CDR3b	AttnLRP	0.034	0.024	-2.82	-2.88

Table 2: AOPCs and LOdds comparison on TCR-XAI and Integrated datasets.

A.6 Maximum vs. Average for Aggregation

High attention weights indicate meaningful interactions and so we used maximum across different cross-attention layers to retain all activated signals. Ablation studies in the table below show that max generally outperforms average, with small exceptions on peptide BRHR and CDR LOdds.

Chain	Mix	ROC-AUC(3.4)	BRHR.25	AOPCs	LOdds
peptide	Max.	0.60	74.3	**0.014**	**-1.52**
peptide	Avg.	0.60	**76.7**	0.013	-1.51
CDR3a	Max.	**0.55**	**79.1**	**0.014**	-3.37
CDR3a	Avg.	0.50	72.6	0.013	**-3.51**
CDR3b	Max.	**0.55**	**79.3**	**0.046**	-3.54
CDR3b	Avg.	0.54	75.3	0.045	**-3.63**

Table 3: Maximum vs. Average for aggregation comparison across chains for ROC-AUC (3.4), BRHR, AOPCs, and LOdds.

A.7 ROC Curves of Residue-Level Importance Scores for Binding Region Identification

We compared QCAI with other methods using ROC curves. We mainly set the distance threshold at 3, 3.4, 4, and 5 Å. 3.4 Å is chosen because it corresponds to the van der Waals diameter between two carbon atoms, implying that residues within this distance are considered to be in contact.

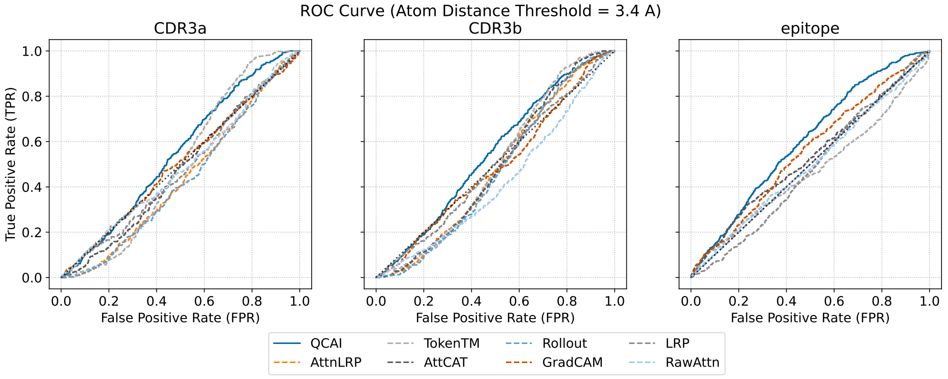

Figure 10: ROC curve comparison of the alpha, beta, and epitope chains between QCAI and other post-hoc methods. The distance threshold is set to 3.4 Å.

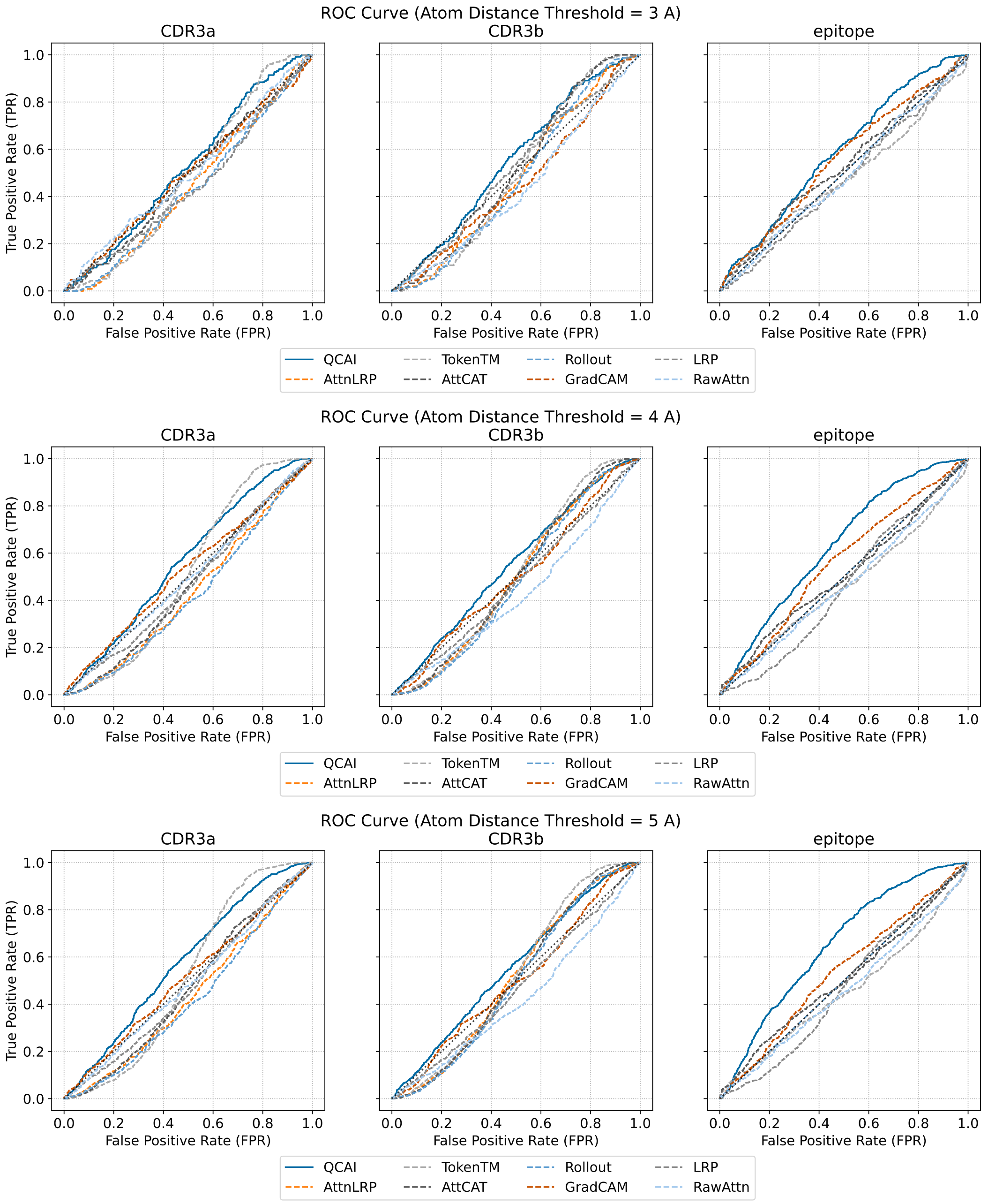

Figure 11: ROC curve comparison of the alpha, beta, and epitope chains between QCAI and other post-hoc methods. The distance thresholds are set to 3, 4, and 5 Å.

A.8 Binding Region Hit Rate

We compare the Binding Region Hit Rate (BRHR) across the TCR α and β chains as well as the epitope region for different explanation methods. Here, HR.t denotes the hit rate calculated based on the top t percentile of importance scores.

Chain	Method	HR.25	HR.30	HR.40	HR.50
epitope	**QCAI (Ours)**	**74.3**(±**24.5**)%	**72.7**(±**24.6**)**%**	**66.4**(±**19.8**)**%**	**55.3**(±**15.7**)**%**
	AttnLRP (Achtibat et al., 2024)	58.4(±29.2)%	60.6(±25.9)%	60.4(±19.8)%	53.7(±15.8)%
	TokenTM (Wu et al., 2024a)	68.5(±29.6)%	66.4(±29.4)%	56.3(±25.9)%	44.4(±20.7)%
	AttCAT (Qiang et al., 2022)	69.1(±30.8)%	66.8(±30.0)%	57.9(±23.9)%	46.5(±18.4)%
	Rollout (Abnar & Zuidema, 2020)	58.4(±29.2)%	60.6(±25.9)%	60.4(±19.8)%	53.7(±15.8)%
	GradCAM (Selvaraju et al., 2017)	72.2(±29.5)%	70.9(±29.3)%	61.9(±24.9)%	50.6(±19.9)%
	LRP (Binder et al., 2016)	60.8(±31.4)%	58.0(±29.8)%	51.7(±21.4)%	42.2(±17.3)%
	RawAttn (Wiegreffe & Pinter, 2019)	68.6(±27.7)%	65.2(±26.0)%	53.7(±23.5)%	42.8(±20.0)%
CDR3a	**QCAI (Ours)**	**79.1**(±**20.1**)**%**	**74.9**(±**19.4**)**%**	**66.7**(±**16.7**)**%**	**55.2**(±**16.6**)**%**
	AttnLRP (Achtibat et al., 2024)	68.5(±24.5)%	63.6(±25.1)%	54.6(±20.9)%	43.0(±19.4)%
	TokenTM (Wu et al., 2024a)	64.4(±30.8)%	60.8(±30.2)%	57.1(±27.1)%	50.1(±20.9)%
	AttCATT (Qiang et al., 2022)	62.7(±25.0)%	60.4(±24.9)%	54.9(±23.4)%	45.0(±19.9)%
	Rollout (Abnar & Zuidema, 2020)	66.6(±25.2)%	61.5(±24.7)%	51.5(±20.7)%	41.1(±18.8)%
	GradCAM (Selvaraju et al., 2017)	66.7(±26.7)%	62.7(±25.5)%	56.1(±20.1)%	46.5(±17.0)%
	LRP (Binder et al., 2016)	66.3(±27.1)%	61.7(±26.6)%	54.9(±21.8)%	46.0(±19.0)%
	RawAttn (Wiegreffe & Pinter, 2019)	65.8(±27.2)%	60.8(±25.3)%	53.1(±22.5)%	44.0(±17.1)%
CDR3b	**QCAI (Ours)**	**79.3**(±**19.0**)**%**	**76.7**(±**18.9**)**%**	**67.7**(±**16.2**)**%**	**56.5**(±**14.9**)**%**
	AttnLRP (Achtibat et al., 2024)	72.6(±25.3)%	66.1(±23.4)%	57.1(±21.8)%	49.5(±17.8)%
	TokenTM (Wu et al., 2024a)	69.5(±27.5)%	66.0(±26.7)%	60.8(±22.0)%	51.6(±18.3)%
	AttCAT (Qiang et al., 2022)	66.9(±25.9)%	64.9(±24.4)%	57.9(±23.2)%	49.3(±19.4)%
	Rollout (Abnar & Zuidema, 2020)	70.4(±25.6)%	64.4(±23.3)%	55.5(±21.5)%	48.3(±18.0)%
	GradCAM (Selvaraju et al., 2017)	71.7(±26.8)%	67.1(±27.1)%	61.0(±24.3)%	48.6(±19.1)%
	LRP (Binder et al., 2016)	61.8(±26.3)%	58.8(±23.1)%	54.6(±20.6)%	45.1(±18.5)%
	RawAttn (Wiegreffe & Pinter, 2019)	69.3(±24.3)%	65.0(±22.2)%	55.9(±19.8)%	44.0(±17.8)%

Table 4: The Binding Region Hit Rate comparison among TCR alpha and beta chains and epitope between various methods. The HR.t denotes the hit rate computed based on the top t percentile.

To consider performance relative to training set similarity we consider the change in BRHR of samples indexed by the Levenshtein distance each input modality to the TULIP training dataset (this approach is also used in the original TULIP paper). In the Table A.8 each cell represents the BRHR of all samples with the minimum Levenshtein distance to the sequences of TULIP training dataset smaller than the given threshold. As a sample's distance between the sequences of TCR-XAI and the sequences of training dataset increases we find that the BRHR score of QCAI on TULIP decreases slightly but remains reliable with the BRHR drop within 0.05, which is small relative to the BRHR difference between QCAI and other methods. This shows that QCAI's performance is preserved even as samples differ from the training set.

Levenshtein Distance (d)	$1 > d$	$2 > d$	$3 > d$	$4 > d$	$5 > d$	$6 > d$	$7 \leq d$
Peptide	.77(.23)	.77(.24)	.80(.23)	.77(.24)	.75(.25)	.74(.25)	.76(.25)
CDR3a	.83(.17)	.81(.18)	.84(.19)	.82(.19)	.79(.20)	.79(.20)	.79(.20)
CDR3b	.83(.20)	.80(.21)	.77(.21)	.78(.21)	.78(.20)	.78(.20)	.78(.20)

Table 5: The change in BRHR for samples indexed by their Levenshtein distance.

To examine how model confidence and prediction outcomes affect BRHR, we further compare BRHR on positive and negative samples for both TULIP and Cross-TCR-Interpreter. Because TULIP provides only relative binding likelihood scores, we perform QCAI analysis on Cross-TCR-Interpreter separately for its predicted positive and negative samples. For TULIP, we additionally set a manual threshold by treating the top 50% scoring pairs as positive and the remaining 50% as negative. The BRHR results shown in Table A.8 indicate that negative samples decreased the quality of explanation comparing to the positive samples.

BRHR	Cross-TCR-Interpreter	Cross-TCR-Interpreter	TULIP	TULIP
**Samples**	Positive	Negative	Positive	Negative
Peptide	.61	.55	.75	.74
CDR3a	.41	.46	.79	.79
CDR3b	.87	.87	.81	.79

Table 6: The BRHR of predicted positive and negative samples.

A.9 TCR-XAI BENCHMARK

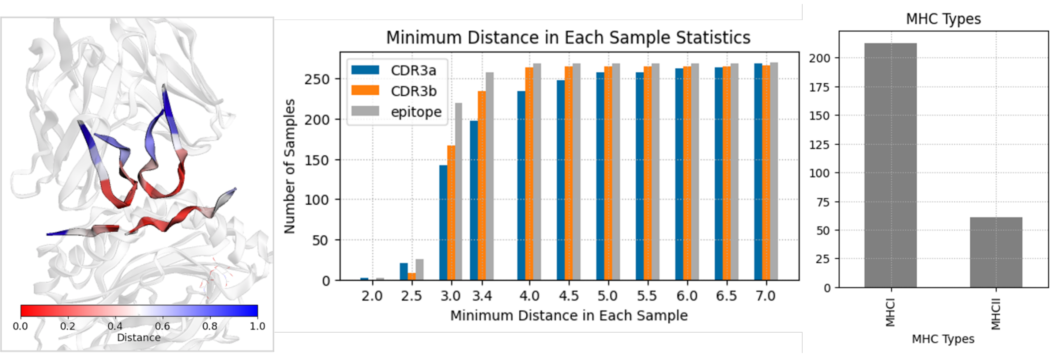

Figure 12: In the example (8TRQ) from the TCR-XAI benchmark, the peptide, CDR3a, and CDR3b regions are highlighted based on their residue-level distances to the nearest interacting residues. Additionally, we report statistics for the minimum distance in each sample and MHC distribution.

We have compiled 274 samples from the STCRDab (Leem et al., 2018) and TCR3d 2.0 (Lin et al., 2025) datasets. Only samples with fully provided CDR3 regions and peptide sequences were selected. Among them, 213 (77.7%) are MHC-I and 61 (22.3%) are MHC-II complexes. For each

sample, we computed the distance from each residue in the CDR3 regions to the nearest atom in the peptide, and vice versa from the peptide residues to the CDR3 regions. The resulting dataset includes both the CDR3 and peptide sequences along with their corresponding residue-level distances. Since the model lacks structural input, we allow a one-residue positional tolerance to account for minor attention shifts. To this end, we smooth each method's output importance scores by convolving them with the kernel $[1/3,\ 1/3,\ 1/3]$ prior to evaluation. The detailed information can be found in Table 9. Compared with the TULIP training dataset, there are 176 distinct epitopes, and none appears in more than 3.3% (9) of the samples.

A.10 Computational Efficiency of QCAI

We evaluate QCAI efficiency based on datasets including VDJdb, IEDB, McPAS-TCR, and TCR-XAI. All evaluations are conducted on CPU (32 E5 cores). QCAI involves pseudo-inverse operations making it more computationally expensive than alternative approaches, but it is still relatively efficient on a per sample basis. For example benchmark sets with thousands of test samples would need on the order of seconds for QCAI evaluation - this is far smaller than what would be needed by practitioners.

Method	TCR-XAI	McPAS-TCR	VDJdb	IEDB
QCAI	2.19 ms	2.18 ms	1.30 ms	1.90 ms
TokenTM	0.11 ms	0.15 ms	0.05 ms	0.11 ms
AttnLRP	0.04 ms	0.02 ms	0.02 ms	0.04 ms

Table 7: Milliseconds per sample for each method across different datasets.

A.11 Ablation Study: QCAI on Cross- vs. Self-Attention

To investigate whether QCAI applied to cross-attention or self-attention contributes more to the final explanation, we compare QCAI applied only to cross-attention, only to self-attention, and to both, using perturbation experiments. Applying QCAI solely to self-attention is equivalent to Rollout. As shown in Figure A.11 and Table A.11, the performance of QCAI on cross-attention alone is comparable to applying it to both cross- and self-attention, and both outperform Rollout. These results indicate that cross-attention is the main contributor to the final explanation and plays a significant role in cross-attention incorporated transformers.

	CDR3a$_{k=4}$		CDR3b$_{k=4}$		Peptide$_{k=7}$	
	LOdds	AOPC	LOdds	AOPC	LOdds	AOPC
QCAI	-3.328	0.014	-3.498	0.045	-1.470	0.013
QCAI (Cross-Attention)	-3.728	0.017	-3.511	0.048	-1.417	0.012
Rollout (Self-Attention)	-2.356	0.022	-2.653	0.032	-0.044	-0.001
AttnLRP	-2.481	0.020	-2.662	0.032	-0.017	0.000
TokenTM	-2.195	0.021	-2.383	0.032	-0.736	0.012

Table 8: Comparison of AOPC and LOdds for QCAI applied to cross-attention only, self-attention only (Rollout), and both.

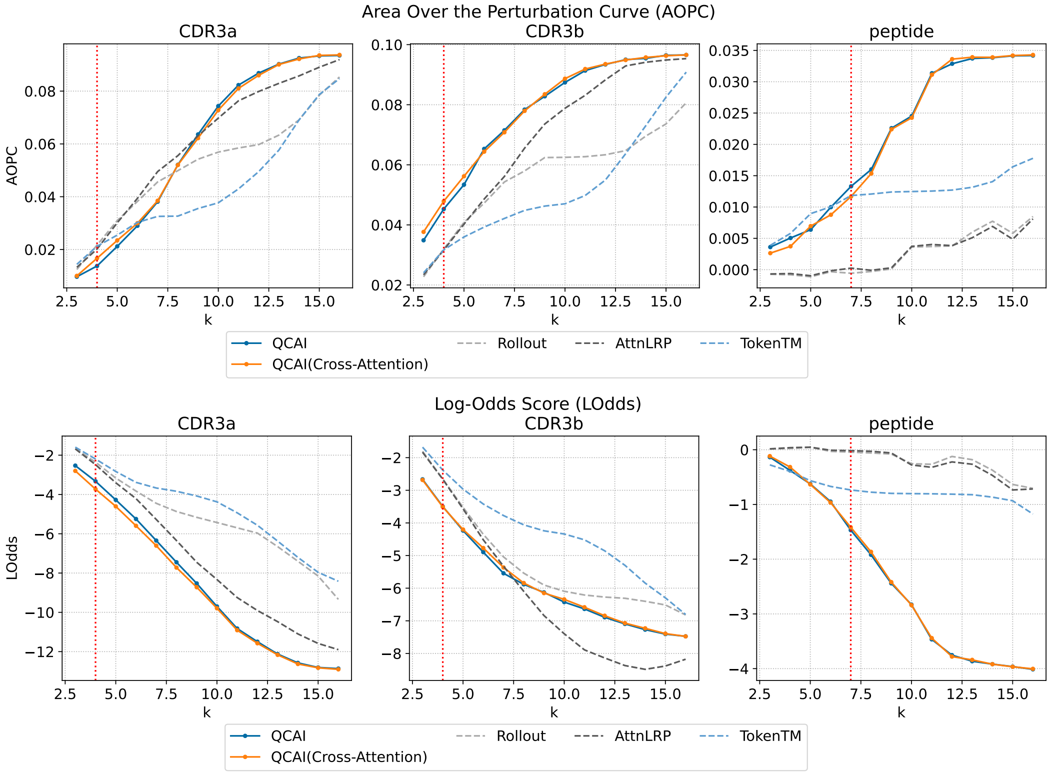

Figure 13: Comparison of AOPC and LOdds for QCAI applied to cross-attention only, self-attention only (Rollout), and both.

A.12 Application of Vision-Language Models

Since QCAI can be applied broadly to cross-attention modules, we illustrate its use in a vision-language model (VLM). For this case study, we employ CLIP Radford et al. (2021), a widely used vision foundation model. CLIP provides separate vision and text encoders with aligned features, so we added a cross-attention layer to fuse image features (as key and value) with text features (as query). We use a subset of the MS-COCO dataset Lin et al. (2014), containing 73,000 images for multi-label classification. The dataset is split 9:1, with 65,700 training samples and 7,300 test samples. The input consists of an image–text pair, where the text is generated following the CLIP paper's recommendation as "a photo of a ..." with the corresponding labels (e.g., "a photo of a cat", "a photo of a couch") Radford et al. (2018). Features extracted via cross-attention are used for label prediction. After 100 epochs of training, the model achieves 94.08% accuracy and 0.9997 ROC-AUC on the test set.

QCAI is then applied to analyze the model. Since each image-text pair has multiple labels, gradients and QCAI are computed for one label at a time. Figure A.12 presents case studies on both training and test samples, demonstrating that QCAI can identify interactions between the two input modalities in cross-attention and highlight the relative importance of the image and text for a given classification label.

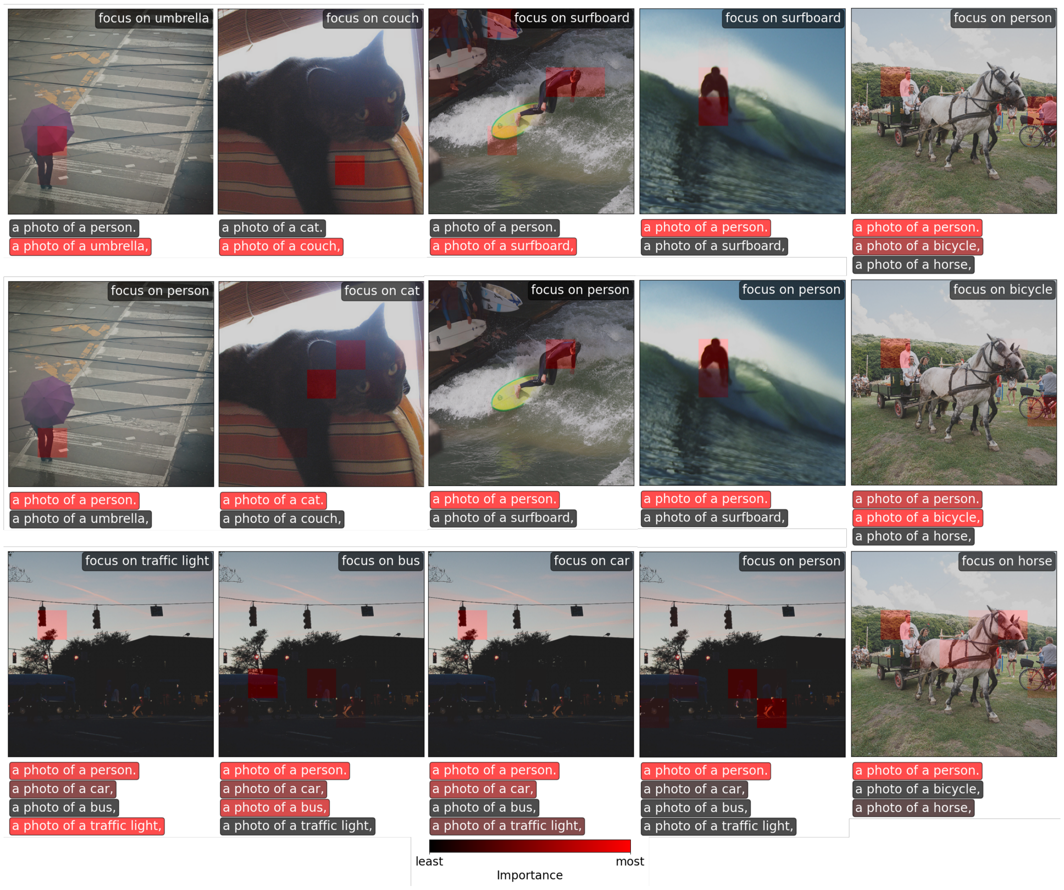

Figure 14: Example of QCAI explaining CLIP with cross-attention.

A.13 TCR-XAI Benchmark Samples

PDB	MHC	Peptide	CDRA3	CDRB3
8TRQ	MHCII	GVYATSSAVRLR	ALGDHSGSWQLI	ASSLRTGANSDYT
4OZI	MHCII	QPFPQPELPYP	LVGDGGSFSGGYNKLI	SAGVGGQETQY
2AK4	MHCI	LPEPLPQGQLTAY	ALSGFYNTDKLI	ASPGLAGEYEQY
5EU6	MHCI	YLEPGPVTV	AVLSSGGSNYKLT	ASSFIGGTDTQY
7PBE	MHCI	YLQPRTFLL	VVNINTDKLI	ASSSANSGELF
4Z7W	MHCII	PSGEGSFQPSQENPQ	AVGETGANNLF	ASSEARRYNEQF
6V18	MHCII	GGYRAPAKAAAT	ALSDSGSFNKLT	ASSLDWGGQNTLY
8EO8	MHCI	LPFDKATIM	AADGGAGSYQLT	SAGPTSGRTDTQY
5WKH	MHCI	GTSGSPIINR	GLGDAGNMLT	ASSLGQGLLYGYT
7T2B	MHCII	ATGLAWEWWRTVYE	ATDKKGGATNKLI	ASSQGGGEQY
7SG1	MHCII	QPFPQPELPYGSGGS	LVGGLARDMR	SVALGSDTGELF
3W0W	MHCI	RFPLTFGWCF	GTYNQGGKLI	ASSGASHEQY
5W1V	MHCI	VMAPRTLIL	AGQPLGGSNYKLT	ASSANPGDSSNEKLF
6AVF	MHCI	APRGPHGGAASGL	LVGEILDNFNKFY	ASSQRQEGDTQY
5NHT	MHCI	ELAGIGILTV	AVGGGADGLT	ASSQGLAGAGELF
3TPU	MHCI	FLSPFWFDI	AVSAKGTGSKLS	ASSDAPGQLY
2P5E	MHCI	SLLLMWIITQC	AVRPLLDGTYIPT	ASSYLGNTGELLF
4P2O	MHCII	PADPLAFFSSAIKGGGGSLV	AALRATGGNNKLT	ASSLNWSQDTQY
7NME	MHCI	QLPRLFPLL	AEPSGNTGKLI	ASSLHHEQY
5JZI	MHCI	KLVALGINAV	AYGEDDKII	ASRRGPYEQY
8I5C	MHCI	VVGAVGVGK	AARDSNYQLI	ASGDTGGYEQY
4E41	MHCII	GELIGILNAAKVPAD	AVDRGSTLGRLY	ASSQIRETQY
6AM5	MHCI	SMLGIGIVPV	AVNFGGGKLI	ASSLSFGTEAF
3E2H	MHCI	QLSPFPFDL	AVSLERPYLT	ASGGGGTLY
3MV8	MHCI	HPVGEADYFEY	AVQDLGTSGSRLT	ASSARSGELF
3KPR	MHCI	EEYLKAWTF	ILPLAGGTSYGKLT	ASSLGQAYEQY
5HYJ	MHCI	AQWGPDPAAA	AMRGDSSYKLI	ASSLWEKLAKNIQY
5KS9	MHCII	APSGEGSFQPSQENPQ	AVALNNNAGNMLT	ASSVAPGSDTQY
7T2D	MHCII	ATGLAWEWWRTVYE	ALSGSARQLT	ASSHREGETQY
1KJ2	MHCI	KVITFIDL	AARYQGGRALI	TCSAAPDWGASAETLY
6RPB	MHCI	SLLMWITQV	AVKSGGSYIPT	ASSYLNRDSALD
6UON	MHCI	GADGVGKSAL	AAAMDSSYKLI	ASSDPGTEAF
1ZGL	MHCII	VHFFKNIVTPRTPG	ALSGGDSSYKLI	ASSLADRVNTEAF
3PQY	MHCI	SSLENFRAYV	ILSGGSNYKLT	ASSFGREQY
4Y19	MHCII	QPLALEGSLQKRG	AASVYAGGTSYGKLT	ASRPRRDNEQF
6AVG	MHCI	APRGPHGGAASGL	LVVDQKLV	ASSGGHTGSNEQF
6V15	MHCII	GGYAPAKAAAT	ALSPSNTNKVV	ASSLDWGVNTLY
4Z7U	MHCII	APSGEGSFQPSQENPQ	ILRDRSNQFY	ASSTTPGTGTETQY
7RM4	MHCI	HMTEVVRHC	ALDIYPHDMR	ASSLDPGDTGELF
4QOK	MHCI	EAAGIGILTV	AVNVAGKST	AWSETGLGTGELF
3PWP	MHCI	LGYGFVNYI	AVTTDSWGKLQ	ASRPGLAGGRPEQY
7N6E	MHCI	YLQPRTFLL	VVNRNNDMR	AGQVTNTGELF
8WUL	MHCI	VVGAVGVGK	AARSSGSWQLI	ASSQDRGDSAHTLY
3DXA	MHCI	EENLLDFVRF	IVWGGYQKVT	ASRYRDDSYNEQF
6RP9	MHCI	SLLMWITQV	ALTRGPGNQFY	ASSSPGGVSTEAF
4MJI	MHCI	TAFTIPSI	ATDDDSARQLT	ASSLTGGGELF
6V19	MHCII	GGYAPAKAAAT	ALSDSSSFSKLV	ASSLDWASQNTLY
7RDV	MHCII	EGRVRVNSAYQS	AASDDNNNRIF	ASGGQSNERLF
3E3Q	MHCI	QLSPFPFDL	AVSDPPPLLT	ASGGGGTLY
4MS8	MHCI	SPAEEAGFFL	AVSAKGTGSKLS	ASSDAPGQLY
2F53	MHCI	SLLMWITQC	AVRPTSGGSYIPT	ASSYVGNTGELF
3QDJ	MHCI	AAGIGILTV	AVNFGGGKLI	ASSLSFGTEAF
6BJ2	MHCI	IPLTEEAEL	ALSHNSGGSNYKLT	ASSFRGGKTQY
3QDM	MHCI	ELAGIGILTV	AGGTGNQFY	AISEVGVGQPQH
5TIL	MHCI	KAPYNFATM	AALYGNEKIT	ASSDAGGRNTLY
6VMX	MHCI	RPPIFIRRL	AFGSSNTGKLI	ASSQDLFTGGYT

Table 9: The samples contained in TCRxAI benchmarks

PDB	MHC	Peptide	CDRA3	CDRB3
7RTR	MHCI	YLQPRTFLL	AVNRDDKII	ASSPDIEQY
8EN8	MHCI	LPFDKSTIM	AADGGAGSYQLT	SAGPTSGRTDTQY
1YMM	MHCII	ENPVVHFFKNIVTP	ATDTTSGTYKYI	SARDLTSGANNEQF
5C07	MHCI	YQFGPDFPIA	AMRGDSSSYKLI	ASSLWEKLAKNIQY
3VXU	MHCI	RFPLTFGWCF	GTYNQGGKLI	ASSGASHEQY
6VQO	MHCI	HMTEVVRHC	AMSGLKEDSSYKLI	ASSIQQGADTQY
1J8H	MHCII	PKYVKQNTLKLAT	AVSESPFGNEKLT	ASSSTGLPYGYT
8ENH	MHCI	LPFEKSTIM	AADGGAGSYQLT	SAGPTSGRTDTQY
2P5W	MHCI	SLLMWITQC	AVRPLLDGTYIPT	ASSYLGNTGELF
3UTT	MHCI	ALWGPDPAAA	AMRGDSSYKLI	ASSLWEKLAKNIQY
7Q99	MHCI	NLSALGIFST	AVNVAGKST	AWSETGLGTGELF
6ZKZ	MHCI	RLPAKAPL	AVTNQAGTALI	ASSYSIRGSRGEQF
4GG6	MHCII	SGEGSFQPSQENP	ILRDGRGGADGLT	ASSVAVSAGTYEQY
6TMO	MHCI	EAAGIGILTV	AVNDGGRLT	AWSETGLGMGGWQ
3QIU	MHCII	ADLIAYLKQATKG	AAEPSSGQKLV	ASSLNNANSDYT
5WKF	MHCI	GTSGSPIVNR	GLGDAGNMLT	ASSLGQGLLYGYT
8SHI	MHCI	VRSRRLRL	ATDALYSGGGADGLT	ASSYSEGEDEAF
5D2N	MHCI	NLVPMVATV	ILDNNNDMR	ASSLAPGTTNEKLF
5KSA	MHCII	QPQQSFPEQEA	AVQFMDSNYQLI	ASSVAGTPSYEQY
6MTM	MHCI	FEDLRVLSF	GTERSGGYQKVT	ASSMSAMGTEAF
2BNQ	MHCI	SLLMWITQV	AVRPTSGGSYIPT	ASSYVGNTGELF
4Z7V	MHCII	SGEGSFQPSQENP	ILRDSRAQKLV	ASSAGTSGEYEQY
2F54	MHCI	SLLMWITQC	AVRPTSGGSYIPT	ASSYVGNTGELF
5BS0	MHCI	ESDPIVAQY	AVRPGGAGPFFVV	ASSFNMATGQY
6CQR	MHCII	RFYKTLRAEQASQ	AFKAAGNKLT	ASSRLAGGMDEQF
5M00	MHCI	KAVANFATM	AALYGNEKIT	ASSDDAAGGGGRNTLY
7N2Q	MHCI	LRVMMLAPF	AVSNFNKFY	ASSVATYSTDTQY
6EQB	MHCI	AAAAGGIIGGIILTV	AVNDGGRLT	AWSETGLGMGGWQQ
4P2R	MHCII	ANGVAFFLTPFKA	AAEASNTNKVV	ASSLNNANSDYT
4P2Q	MHCII	ADGLAYFRSSFKGG	AAEASNTNKVV	ASSLNNANSDYT
8DNT	MHCI	LLLDRLNQL	AVREGAQKLV	ASSLDLGADEQF
5E6I	MHCI	GILGFVFTL	AGPGGSSNTGKLI	ASSLIYPGELF
5TJE	MHCI	KAVYNFATM	AALYGNEKIT	ASSDAGGRNTLY
2J8U	MHCI	ALWGFFPVL	ALFLASSSFSKLV	ASSDWVSYEQY
1LP9	MHCI	ALWGFFPVL	ALFLASSSFSKLV	ASSDWVSYEQY
3KPS	MHCI	EEYLQAFTY	ILPLAGGTSYGKLT	ASSLGQAYEQY
2BNR	MHCI	SLLMWITQC	AVRPTSGGSYIPT	ASSYVGNTGELF
5W1W	MHCI	VMAPRTLVL	AGQPLGGSNYKLT	ASSANPGDSSNEKLF
6CQL	MHCII	RFYKTLRAEQASQ	AFKAAGNKLT	ASSRLAGGMDEQF
5C09	MHCI	YLGGPDFPTI	AMRGDSSYKLI	ASSLWEKLAKNIQY
4MXQ	MHCI	SPAPRPLDL	AVSAKGTGSKLS	ASSDAPGQLY
3SJV	MHCI	FLRGRAYGL	VVRAGKLI	ASGQGNFDIQY
1QRN	MHCI	LLFGYAVYV	AVTTDSWGKLQ	ASRPGLAGGRPEQY
3KXF	MHCI	LPEPLPQGQLTAY	ALSGFYNTDKLI	ASPGLAGEYEQY
5C0A	MHCI	MVWGPDPLYV	AMRGDSSYKLI	ASSLWEKLAKNIQY
7N2N	MHCI	TRLALIAPK	AVLSPVQETSGSRLT	ASSVGLFSTDTQY
8ES9	MHCI	GVYDGREHTV	AVQPLNAGNNRKLI	SAREWGGTEAF
2NX5	MHCI	EPLPQGQLTAY	AVQASGGSYIPT	ATGTGDSNQPQH
1G6R	MHCI	SIYRYYGL	AVSGFASALT	ASGGGGTLY
8GVB	MHCI	RYPLTFGW	AVGFTGGGNKLT	ASSDRDRVPETQY
8TRL	MHCII	EIFDSGNPTGEV	IVNPANTGNQFY	ASRRDYFSYEQY
5D2L	MHCI	NLVPMVATV	AFITGNQFY	ASSQTQLWETQY
5WLG	MHCI	SQLLNAKYL	ATVYAQGLT	ASSDWGDTGQLY
5NMG	MHCI	SLFNTIAVL	AVRTNSGYALN	ASSDTVSYEQY
7DZM	MHCI	TPQDLNTML	IVRGLNNAGNMLT	ASSLGIDAIY
7BYD	MHCI	GGAI	LVGGGGYVLT	ASSQDLGAGEVYEQY
5HHO	MHCI	GILEFVFTL	AGAGSQGNLI	ASSIRSSYEQY

Table 10: The samples contained in TCRxAI benchmarks (continue table 1)

PDB	MHC	Peptide	CDRA3	CDRB3
1QSE	MHCI	LLFGYPRYV	AVTTDSWGKLQ	ASRPGLAGGRPEQY
3RGV	MHCI	WIYVYRPMGCGGS	AANSGTYQR	ASGDFWGDTLY
2E7L	MHCI	QLSPFPFDL	AVSHQGRYLT	ASGGGGTLY
3MBE	MHCII	GAMKRHGLDNYRGYSLG	AAEDGGSGNKLI	ASSWDRAGNTLY
5M01	MHCI	KAPANFATM	AALYGNEKIT	ASSDDAAGGGGRRNTLY
5SWZ	MHCI	ASNENMETM	AASETSGSWQLI	ASSRDLGRDTQY
5NMF	MHCI	SLYNTIATL	AVRTNSGYALN	ASSDTVSYEQY
8GVI	MHCI	RYPLTFGW	AVVFTGGGNKLT	ASSLRDRVPETQY
7N5C	MHCI	SSLCNFRAYV	ILSGGCCNYKLT	ASSFGREQY
8TRR	MHCII	GVYATSSAVRLR	ALGDTGNYKYV	ASSAVNSGNTLY
4OZG	MHCII	APQPELPYPQPG	IVLGGADGLT	ASSFRFTDTQY
4OZH	MHCII	APQPELPYPQPGS	IVWGGATNKLI	ASSVRSTDTQY
2OL3	MHCI	SQYYYNSL	AMRGDYGGSGNKLI	TCSADRVGNTLY
7QPJ	MHCI	GLYDGMEHL	AVRGTGRRALT	ASSFATEAF
3VXM	MHCI	RFPLTFGWCF	AVGAPSGAGSYQLT	ASSPTSGIYEQY
6EQA	MHCI	AAAAGGIIGGIILTV	AVNVAGKST	AWSETGLGTGELF
2YPL	MHCI	KAFSPEVIPMF	AVSGGYQKVT	ASTGSYGYT
7RK7	MHCI	YMDGTMSQV	LVALNYGGSQGNLI	AISPTEEGGLIFPGNTIY
8WTE	MHCI	VVGAVGVGK	AARSSGSWQLI	ASSQDRGDSAETLY
3UTS	MHCI	ALWGPDPAAA	AMRGDSSYKLI	ASSLWEKLAKNIQY
1QSF	MHCI	LLFGYPVAV	AVTTDSWGKLQ	ASRPGLAGGRPEQY
1OGA	MHCI	GILGFVFTL	AGAGSQGNLI	ASSSRSSYEQY
2GJ6	MHCI	LLFGKPVYV	AVTTDSWGKLQ	ASRPGLAGGRPEQY
3QDG	MHCI	ELAGIGILTV	AVNFGGGKLI	ASSLSFGTEAF
2VLR	MHCI	GILGFVFTL	AGAGSQGNLI	ASSSRASYEQY
7NA5	MHCI	YGFRNVVHI	AVSNYNVLY	ASSQEPGGYAEQF
8CX4	MHCI	LRVMMLAPF	AVNSPGSGAGSYQLT	ASSVGTYSTDTQY
4PRI	MHCI	HPVGEADYFEY	AVQDLGTSGSRLT	ASSARSGELF
8YE4	MHCI	NYNYLYRLF	VVNAHSGAGSYQLT	ASSETGGYEQY
5M02	MHCI	KAPFNFATM	AALYGNEKIT	ASSDAGGRNTLY
2CKB	MHCI	EQYKFYSV	AVSGFASALT	ASGGGGTLY
3TFK	MHCI	QLSDVPMDL	AVSAKGTGSKLS	ASSDAPGQLY
7N2S	MHCI	TRLALIAPK	AVSLGTGAGSYQLT	ASSVGLYSTDTQY
5KSB	MHCII	GPQQSFPEQEA	AVQASGGSYIPT	ASSNRGLGTDTQY
2UWE	MHCI	ALWGFFPVL	ALFLASSSFSKLV	ASSDWVSYEQY
7Q9A	MHCI	LLLGIGILVL	AVNVAGKST	AWSETGLGTGELF
5C08	MHCI	RQWGPDPAAV	AMRGDSSYKLI	ASSLWEKLAKNIQY
3HG1	MHCI	ELAGIGILTV	AVNVAGKST	AWSETGLGTGELF
8I5D	MHCI	VVGAVGVGK	AASSGSWQLI	ASSLEGTVEETLY
5JHD	MHCI	GILGFVFTL	AWGVNAGGTSYGKLT	ASSIGVYGYT
7JWJ	MHCI	ASNENMETM	AAVTGNTGKLI	ASSRGTIHSNTEVF
4MNQ	MHCI	ILAKFLHWL	AVDSATALPYGYI	ASSYQGTEAF
6PY2	MHCII	APFSEQEQPVLG	ASPQGGSEKLV	ASSSGGWGGGTEAF
7DZN	MHCI	TPQDLNTML	IVRGLNNAGNMLT	ASSLGIDAIY
4EUP	MHCI	ALGIGILTV	AVSGGGADGLT	ASSFLGTGVEQY
7N1E	MHCI	RLQSLQTYV	ALSGFNNAGNMLT	ASSLGGAGGADTQY
3QEQ	MHCI	AAGIGILTV	AGGTGNQFY	AISEVGVGQPQH
2IAN	MHCII	GELIGTLNAAKVPAD	AALIQGAQKLV	ASTYHGTGY
2VLJ	MHCI	GILGFVFTL	AGAGSQGNLI	ASSSRSSYEQY
6CQN	MHCII	RFYKTLRAEQASQ	AFKAAGNKLT	ASSGLAGGMDEQF
3VXR	MHCI	RYPLTFGWCF	AVRMDSSYKLI	ASSSWDTGELF
7NMG	MHCI	LWMRLLPLL	AEPSGNTGKLI	ASSLHHEQY
3D3V	MHCI	LLFGPVYV	AVTTDSWGKLQ	ASRPGLAGGRPEQY
5ISZ	MHCI	GILGFVFTL	AFDTNAGKST	ASSIFGQREQY
6U3N	MHCII	APMPMPELPYP	AVGAGSNYQLI	ASSLEGQGASEQF
6RSY	MHCI	RMFPNAPYL	IGGGTTSGTYKYI	ASSLGFGRDVMR
4MVB	MHCI	QPAEGGFQL	AVSAKGTGSKLS	ASSDAPGQLY

Table 11: The samples contained in TCRxAI benchmarks (continue table 2)

PDB	MHC	Peptide	CDRA3	CDRB3
1MI5	MHCI	FLRGRAYGL	ILPLAGGTSYGKLT	ASSLGQAYEQY
3VXS	MHCI	RYPLTLGWCF	AVRMDSSYKLI	ASSSWDTGELF
7OW5	MHCI	VVVGAGGVGK	AMSVPSGDGSYQFT	ASKVGPGQHNSPLH
8GVG	MHCI	RFPLTFGW	AVGFTGGGNKLT	ASSDRDRVPETQY
2VLK	MHCI	GILGFVFTL	AGAGSQGNLI	ASSSRSSYEQY
8GOM	MHCI	RLQSLQTYV	ASSGNTPLV	ASTWGRASTDTQY
1D9K	MHCII	GNSHRGAIEWEGIESG	AATGSFNKLT	ASGGQGRAEQF
6CQQ	MHCII	RFYKTLRAEQASQ	AFKAAGNKLT	ASSRLAGGMDEQF
5HHM	MHCI	GILGLVFTL	AGAGSQGNLI	ASSSSRSSYEQY
4N0C	MHCI	MPAGRPWDL	AVSAKGTGSKLS	ASSDAPGQLY
5C0B	MHCI	RQFGPDFPTI	AMRGDSSYKLI	ASSLWEKLAKNIQY
7PB2	MHCI	VVVGADGVGK	ALSGPSGAGSYQLT	ASSYGPGQHNSPLH
1MWA	MHCI	EQYKFYSV	AVSGFASALT	ASGGGGTLY
4QRP	MHCI	HSKKKCDEL	ALSDPVNDMR	ASSLRGRGDQPQH
6RPA	MHCI	SLLMWITQV	AVRDINSGAGSYQLT	SVGGSGGADTQY
7N5P	MHCI	SSLCNFRAYV	ILSGGSNYKLT	ASSFFGREQY
7N1F	MHCI	YLQPRTFLL	AVNRDDKII	ASSPDIEQY
5C0C	MHCI	RQFGPDWIVA	AMRGDSSYKLI	ASSLWEKLAKNIQY
6D78	MHCI	AAGIGILTV	AVNFGGGKLI	ASSWSFGTEAF
4JFF	MHCI	ELAGIGILTV	AVNDGGRLT	AWSETGLGMGGWQ
4N5E	MHCI	VPYMAEFGM	AVSAKGTGSKLS	ASSDAPGQLY
4JRX	MHCI	LPEPLPQGQLTAY	ALSGFYNTDKLII	ASPGETEAF
7NMF	MHCI	QLPRLFPLL	AEPSGNTGKLI	ASSLHHEQY
3QIW	MHCII	ADLIAYLEQATKG	AAEPSSGQKLV	ASSLNNANSDYT
6ZKX	MHCI	RLPAKAPLLGCG	AVTNQAGTALI	ASSYSIRGSRGEQF
1NAM	MHCI	RGYVYQGL	AMRGDYGGSGNKLI	TCSADRVGNTLY
8PJG	MHCII	PKYVKQNTLKLAR	AVSEQDDKII	ATSDESYGYT
8VCX	MHCII	GQVELGGGPGAESCQ	IVSHNAGNMLT	ASSLERETQY
5YXU	MHCI	KLVALGINAV	AYGEDDKII	ASRRGSAELY
3O4L	MHCI	GLCTLVAML	AEDNNARLM	SARDGTGNGYT
7SG2	MHCII	QPFPQPEQPFPGS	LVGGLARDMR	SVALGSDTGELF
8GON	MHCI	RLQSLQIYV	ASSGNTPLV	ASTWGRASTDTQY
2JCC	MHCI	ALWGFFPVL	ALFLASSSFSKLV	ASSDWVSYEQY
6G9Q	MHCI	KAPYDYAPI	AALYGNEKIT	ASSDAGGRNTLY
6DKP	MHCI	ELAGIGILTV	AVNFGGGKLI	ASSWSFGTEAF
5NQK	MHCI	ELAGIGILTV	AGGGGADGLT	ASSQGLAGAGELF
2PYE	MHCI	SLLMWITQC	AVRPLLDGTYIPT	ASSYLGNTGELF
6R2L	MHCI	SLSKILDTV	AVGGNDWNTDKLI	ASSPLDVSISSYNEQF
2OI9	MHCI	QLSPFPFDL	AVSGFASALT	ASGGGGTLY
8F5A	MHCI	TSTLQEQIGW	AVTLNNNAGNMLT	ASSVGGTEAF
7Z50	MHCII	LQTLALEVEDDPC	AASVRNYKYV	ASSRQGQNTLY
6BGA	MHCII	YVVVPD	AALRATGGNNKLT	ASSLNWSQDTQY
3MV7	MHCI	HPVGEADYFEY	AVVQDLGTSGSRLT	ASSARSGELF
8VD2	MHCII	GQVELGGGTPIESC	IVRVAIEGSQGNLI	ASSLRRGDTIY
8VCY	MHCII	GQVELGGGSSPETCI	IVSHNAGNMLT	ASSLERETQY
5YXN	MHCI	KLVALGINAV	AYGEDDKII	ASRRGPYEQY
5E9D	MHCI	ELAGIGILTV	AVTKYSWGKLQ	ASRPGWMAGGVELY
6AMU	MHCI	MMWDRGLGMM	AVNFGGGKLI	ASSLSFGTEAF
5BRZ	MHCI	EVDPIGHLY	AVRPGGAGPFFVV	ASSFNMATGQY
3TJH	MHCI	SPLDSLWWI	AVSAKGTGSKLS	ASSDAPGQLY
3H9S	MHCI	MLWGYLQYV	AVTTDSWGKLQ	ASRPGLAGGRPEQY
4PRP	MHCI	HPVGQADYFEY	AVQDLGTSGSRLT	ASSARSGELF
5IVX	MHCI	RGPGRAFVTI	AASASFGDNSKLI	ASSLGHTEVF
4Y1A	MHCII	LQPLALEGSLQKRG	AASSSAGGTSYGKLT	ASRPRDPVTQY
2IAM	MHCII	GELIGILNAAKVPAD	AALIQGAQKLV	ASTYHGTGY
6U3O	MHCII	AVVQSELPYPEGS	IAFQGAQKLV	ASSFRALAADTQY

Table 12: The samples contained in TCRxAI benchmarks (continue table 3)

PDB	MHC	Peptide	CDRA3	CDRB3
6V0Y	MHCII	GGYAPAKAAAT	ALSDSGSFNKLT	ASSLDWGGQNTLY
5NME	MHCI	SLYNTVATL	AVRTNSGYALN	ASSDTVSYEQY
7T2C	MHCII	TGLAWEWWRTVY	LVGDTGFQKLV	SARDPGGGGSSYEQY
2PXY	MHCII	RGGASQYRPSQ	ALSENYGNEKIT	ASGDASGAETLY
4G9F	MHCI	KRWIIMGLNK	AMRDLRDNFNKFY	ASREGLGGTEAF
3QIB	MHCII	ADLIAYLKQATKG	AALRATGGNNKLT	ASSLNWSQDTQY
4G8G	MHCI	KRWIILGLNK	AMRDLRDNFNKFY	ASREGLGGTEAF
7N2O	MHCI	LRVMMLAPF	AVLSPVQETSGSRLT	ASSVGLFSTDTQY
5TEZ	MHCI	GILGFVFTL	AASFIIQGAQKLV	ASSLLGGWSEAF
3D39	MHCI	LLFGPVYV	AVTTDSWGKLQ	ASRPGLAGGRPEQY
6ZKW	MHCI	RLPAKAPLL	AVTNQAGTALI	ASSYSIRGSRGEQF
4FTV	MHCI	LLFGYPVYV	AVTTDSWGKLQ	ASRPGLMSAQPEQY
6PX6	MHCII	APFSEQEQPVLG	AVHTGARLM	ASSHGASTDTQY
6V1A	MHCII	GGYRAPAKAAAT	ALSDSSSFSKLV	ASSLDWASQNTLY
1U3H	MHCII	SRGGASQYRPSQ	AASANSGTYQR	ASGDAGGGYEQY
7N4K	MHCI	SSLENFRRAYV	ILSGGSNYKLT	ASSFFGREQY
2Z31	MHCII	RGGASQYRPSQ	ALSENYGNEKIT	ASGDASGGNTLY
2ESV	MHCI	VMAPRTLIL	IVVRSSNTGKLI	ASSQDRDTQY
5EUO	MHCI	GILGFVFTL	AGAIGPSNTGKLI	ASSIRSSYEQY
4JFD	MHCI	ELAAIGILTV	AVNDGGRLT	AWSETGLGMGGWQ
6ZKY	MHCI	RLPAKAPL	AVTNQAGTALI	ASSYSIRGSRGEQF
6TRO	MHCI	GVYDGREHTV	VVNHSGGSYIPT	ASSFLMTSGDPYEQY
7N2P	MHCI	GQVMVVAPR	AVSNFNKFY	ASSVATYSTDTQY
7R80	MHCI	QASQEVKNW	AQLNQAGTALI	ASSYGTGINYGYT
1BD2	MHCI	LLFGYPVYV	AAMEGAQKLV	ASSYPGGGFYEQY
4L3E	MHCI	ELAGIGILTV	AVNFGGGKLI	ASSWSFGTEAF
7PHR	MHCI	YLEPGPVTV	ATDGSTPMQ	ASSWGAPYEQY
3FFC	MHCI	FLRGRAYGL	AMREDTGNQFY	ASSFTWTSGGATDTQY
4JRY	MHCI	LPEPLPQGQLTAY	AVGGGSNYQLI	ASSRTGSTYEQY
5SWS	MHCI	ASNENMETM	AASEGSGSWQLI	ASSAGLDAEQY
6UZ1	MHCI	LLFGYPVYV	AVTTDRSGKLQ	ASRPGAAGGRPELY
1FO0	MHCI	INFDFNTI	AMRGDYGGSGNKLI	TCSADRVGNTLY
7JWI	MHCI	ASNENMETM	AASETSGSWQLI	ASSRDLGRDTQY
8D5Q	MHCI	HPGSVNEFDF	ALGDPTGANTGKLT	TCSAGRGGYAEQF
6VRM	MHCI	HMTEVVRHC	VVQPGGYQKVT	ASSEGLWQVGDEQY
7N2R	MHCI	TRLALIAPK	AVSNFNKFY	ASSVATYSTDTQY
1FYT	MHCII	PKYVKQNTLKLAT	AVSESPFGNEKLT	ASSSTGLPYGYT
3QFJ	MHCI	LLFGFPVYV	AVTTDSWGKLQ	ASRPGLAGGRPEQY
3GSN	MHCI	NLVPMVATV	ARNTGNQFY	ASSPVTGGIYGYT
6V13	MHCII	GGYRAPAKAAAT	ALSPSNTNKVV	ASSLDWGVNTLY
7OW6	MHCI	VVVGADGVGK	AMSVPSGDGSYQFT	ASKVGPGQHNSPLH
4OZF	MHCII	APQPELPYPQPGS	IAFQGAQKLV	ASSFRALAADTQY
4JFE	MHCI	ELAGIGALTV	AVNDGGRLT	AWSETGLGMGGWQ
3MV9	MHCI	HPVGEADYFEY	AVQDLGTSGSRLT	ASSARSGELF
6Q3S	MHCI	SLLMWITQV	AVRPTSGGSYIPT	ASSYVGNTGELF
5MEN	MHCI	ILAKFLHWL	AVDSATSGTYKYI	ASSYQGTEAF
1AO7	MHCI	LLFGYPVYV	AVTTDSWGKLQ	ASRPGLAGGRPEQY
4H1L	MHCII	QHIRCNIPKRISA	AVGASGNTGKLI	ASSLRDGYTGELF

Table 13: The samples contained in TCRxAI benchmarks (continue table 4)

B REPRODUCIBILITY STATEMENT

The source code is publicly available at our project website (`https://qcai.jiarui.li/`) and on GitHub (`https://github.com/Jiarui0923/QCAI`).

C LARGE LANGUAGE MODEL USAGE STATEMENT

We employed large language models (LLMs), primarily ChatGPT, in two limited ways:

- as a coding assistant, and
- for polishing written text.

Coding Assistant LLMs were consulted to clarify documentation, organize API references, and suggest debugging strategies. All code, documentation, and fixes obtained were manually reviewed and verified by the authors.

Polishing Article LLMs were used only to refine the clarity and style of sentences written by the authors and to format tables from raw data. No raw text or substantive content was generated by LLMs. All refined content was manually checked and further revised by the authors.
